# Changes in microglial morphologies during brain aging in common marmosets

**DOI:** 10.1007/s00429-026-03082-z

**Published:** 2026-02-17

**Authors:** Kimberley A. Phillips, Savannah K. Boyen, Katelin X. Oliveira, Reagan Meredith, Chet C. Sherwood

**Affiliations:** 1 Department of Psychology, Trinity University, San Antonio, TX USA; 2 Southwest National Primate Research Center, Texas Biomedical Research Institute, San Antonio, TX USA; 3 Department of Anthropology and Center for the Advanced Study of Human Paleobiology, The George Washington University, Washington, DC USA

**Keywords:** Aging, Neurodegeneration, Microglial dysfunction, Iba1

## Abstract

Microglia are critical modulators of neuronal activity, regulating brain function through removal of dying neurons, pruning non-functional synapses, and producing ligands that support neuronal survival. Microglial morphologies change with aging, shifting from a homeostatic, surveillant state (ramified morphology) to a more reactive state (intermediate, amoeboid, and dystrophic morphology). Common marmosets (*Callithrix jacchus*) are a valuable translational nonhuman primate model for aging, and studies of neuroinflammation and neurodegenerative disease. These small, genetically diverse platyrrhine primates are considered aged at 7 years, have a maximum potential lifespan of 12+ years, and display age-related changes in motor, sensorimotor, and cognitive function. Here we quantified microglial morphologies in 24 aged marmosets (7–18 years) in the dorsolateral prefrontal cortex (dlPFC, Brodmann’s area 9), hippocampal regions CA1 and CA3, and the entorhinal cortex (ENT) via immunohistochemistry against Iba1. The proportion of ramified microglia showed a significant negative correlation with age in dlPFC, CA1, and CA3, and the proportion of dystrophic microglia showed a significant positive correlation with age in all four regions examined. Males displayed a significantly higher proportion of dystrophic microglia than females in CA1. Sex did not significantly affect the proportion of microglia morphologies in any other brain region. A significant interaction between age and sex was found for the proportion of intermediate microglia in ENT. Overall, these data suggest brain aging in marmosets from middle-age to older adulthood is characterized by an increase in dystrophic microglia, and age-related changes in microglial morphology manifest differently in male and female marmosets in hippocampal regions.

**Supplementary Information:**

The online version contains supplementary material available at 10.1007/s00429-026-03082-z.

## Introduction

Microglia are the resident macrophages of the central nervous system and key components of the brain’s immune defense system. They maintain the integrity of neuronal circuitry in the brain by phagocytizing apoptotic or necrotic cells, and engulfing synaptic, axonal, and myelin debris. During aging, microglia undergo morphological alterations which are related to their activity state, inflammatory response, and overall function (Streit et al 2004; Xue and Streit 2011). Microglial phenotypes are varied and have been historically categorized as resting/surveilling, anti-inflammatory, and pro-inflammatory (though accumulating evidence indicates there are additional phenotypes) (Butovsky and Weiner 2018; Martinez and Gordon 2014). The resting/surveilling phenotype is observed under healthy conditions. Aging microglia shift from a homeostatic, surveillant state (ramified morphology) to a more reactive state (intermediate and amoeboid morphology). The dystrophic/senescent phenotype is associated with aging and neurodegenerative disease; microglia reach an end stage of dysfunction, undergoing dystrophy in regards to their morphology (Rodriguez-Callejas et al. 2016; Streit et al. 2004; Shahidehpour et al. 2021). Dystrophic microglia lose their function as cytoplasmic disruption occurs (Tischer et al. 2016), making neurons more vulnerable to toxic substances and viruses. Dystrophic microglia are associated with senescence and increased markers of activation, and are most often seen in very aged brains and in neurodegenerative disease, reflecting functional impairment and heightened responsiveness to brain insults (Spittau 2017; Antignano et al. 2023). Furthermore, microglial senescence is strongly linked to cognitive impairment, especially in the context of brain aging and neurodegeneration, by promoting chronic inflammation, reducing the clearance of neurotoxic proteins, and facilitating neurodegenerative pathology progression (Ng et al. 2023; Rim et al. 2024).

Only a few studies have examined changes in microglial morphologies with age in nonhuman primates, though a pattern of increased microglial activation and densities has emerged. Activated microglia density and morphology in aged chimpanzees (*Pan troglodytes*) was associated with amyloid beta (Aβ) plaque deposition, which correlated with increased microglial activation and a shift toward an intermediate morphology in the hippocampus (Edler et al. 2018). Aged rhesus macaque monkeys (*Macaca mulatta*) had increased microglial densities in gray matter of the frontal lobe and retrosplenial cortex compared to younger adults (Robillard et al. 2016; Gray et al. 2023), and aged rhesus had a 44% increase in microglial density in the visual cortex compared to younger animals (Peters et al. 1991). However, as other studies did not detect age-associated differences in activated microglial densities in visual cortex, substantia nigra pars compacta, and the ventral tegmental area in rhesus monkeys (Kanaan et al. 2010; Peters et al. 2008), such changes may be region-specific. Rodriguez-Callejas et al. (2019) quantified resting, active, and dystrophic microglia densities in the dorsal hippocampus of male common marmosets (*Callithrix jacchus*) ranging from 2–18 years. While differences in total microglia number were not detected across the ages, age-dependent changes were found. Resting microglia were the highest in adolescent marmosets compared to old and aged marmosets; aged animals had significantly decreased activated microglial; and aged animals had the highest frequency of dystrophic microglia.

In this study, we assessed microglial morphology in a relatively large sample of aged and very aged common marmosets. We were particularly interested in whether marmosets exhibited sex differences in microglial morphology in regions underlying cognitive abilities: the dorsolateral prefrontal cortex (dlPFC, Brodmann’s area 9), hippocampal regions CA1 and CA3, and the entorhinal cortex (ENT). Common marmosets are increasingly used as models of aging and neurodegenerative disease (Sukoff Rizzo et al. 2023; Perez-Cruz and Rodriguez-Callejas 2023). The brains of aged marmosets spontaneously present amyloid-ß aggregates, dystrophic microglia, tau hyperphosphorylation, and loss of calbindin positive cells (Rodriguez-Callejas et al. 2016; Freire-Cobo et al. 2023; Perez-Cruz and Rodriguez-Callejas 2023; Sharma et al. 2019) and show age-related cognitive impairment (Phillips et al. 2019; LaClair et al. 2019; Workman et al. 2019; Glavis-Bloom et al. 2022; Vanderlip et al. 2023). Accumulating evidence indicates cognitively impaired aged marmosets show signs of neurodegeneration and accelerated brain aging (Freire-Cobo et al. 2023), with cognitive decline in aged marmosets associated with increased astrogliosis and heightened phagocytic activity of microglia. Female marmosets may exhibit earlier and more pronounced cognitive decline than males (Rothwell et al. 2022). Therefore, understanding the natural aging process, including changes in microglial morphologies in regions associated with cognition, is critical in understanding the role of microglia in neurological aging and neurodegeneration, and furthering our understanding of marmosets as a potential model of brain aging.

## Method

### Specimens and sample processing

**Fig. 1:**
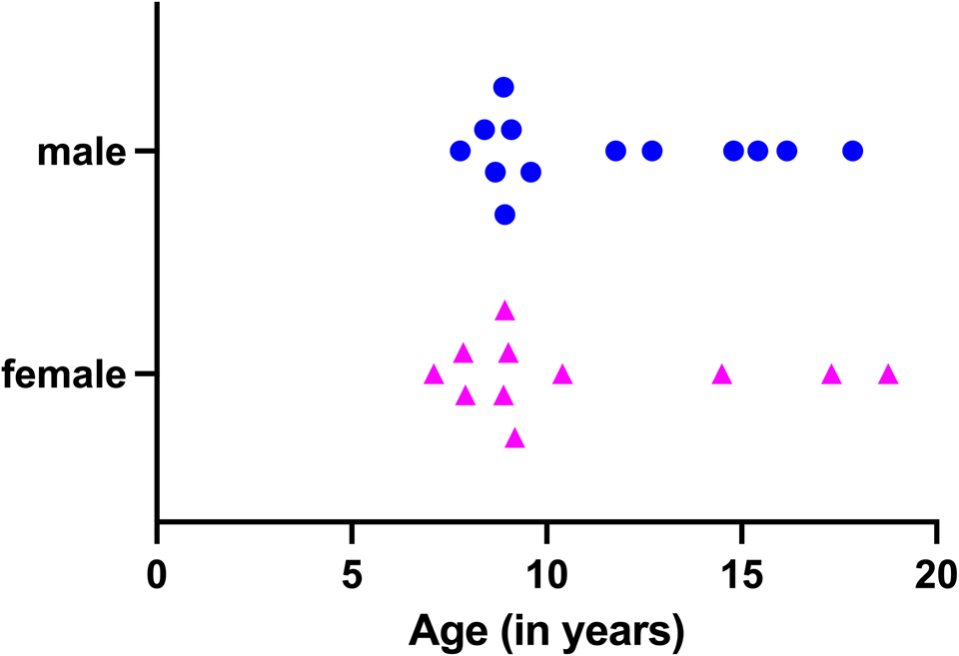
Age distribution (in years) of the male and female marmoset samples used in this study. Females are colored lavender; males are colored plum

The sample was comprised of brains from 24 common marmosets (male *n* = 13, female *n* = 11), ranging from 7 to 18 years (Fig. 1). All individuals were housed in laboratory settings (pair- or family-housed) and were either part of a breeding group or involved in non-invasive studies. Animals were housed at the Southwest National Primate Research Center, Texas Biomedical Research Institute, San Antonio, USA and maintained in accordance with the *Guide for the Care and Use of Laboratory Animals*. Marmosets are considered aged at 7 years and very aged or geriatric at 10 years (Geula et al. 2002; Ross 2019). Brains were collected opportunistically following euthanasia for health reasons. The brain was removed at necropsy, bisected, and the right hemisphere was immersion fixed into 4% paraformaldehyde. After a fixation period of no less than 7 days, the brain was transferred into PBS + 0.1% sodium azide and stored at 4°. None of the brains showed gross abnormalities or pathology on inspection.

The brains were sent to NeuroScience Associates (Knoxville, TN, USA) for sectioning and histological processing. Brains were treated overnight with 20% glycerol and 2% dimethylsulfoxide to prevent freeze-artifacts. The specimens were then embedded in a gelatin matrix using MultiBrain®/ MultiCord® Technology (NeuroScience Associates, Knoxville, TN). The blocks were rapidly frozen, after curing by immersion in 2-Methylbutane chilled with crushed dry ice and mounted on a freezing stage of an AO 860 sliding microtome. The MultiBrain®/MultiCord® blocks were sectioned coronally at 40 µm thickness. All sections were cut through the entire length of the specimen segment and collected sequentially into series of 24 containers. All containers contained Antigen Preserve solution (50% PBS pH7.0, 50% Ethylene Glycol, 1% Polyvinyl Pyrrolidone); no sections were discarded.

### Thionin-Nissl staining

Series of the free-floating coronal sections were used for histology, using the Thionin-Nissl stain to label neurons in the brain. Every 10th free-floating section was used, mounted on gelatin coated glass slides in a serial order, rostral to caudal. The slides were air dried and underwent the histology protocol in the following sequence: 95% ethanol, 95% ethanol/Formaldehyde; 95% ethanol, Chloroform/Ether/absolute ethanol (8:1:1), 95% ethanol; 10% HCl/ethanol, 95% ethanol, 70% ethanol, deionized water, Thionin (0.05% Thionin/acetate buffer, pH 4.5), deionized water, 70% ethanol, 95% ethanol, Acetic Acid/ethanol, 95% ethanol, 100% ethanol, 100% ethanol, 1:1 100% ethanol/xylene, xylene, xylene, and then coverslipped.

### Immunohistochemistry

Every 20th free floating section was immunohistochemically stained against the ionized calcium-binding adaptor molecule 1 (Iba1) using the avidin–biotin peroxidase method. Iba1, the rabbit monoclonal antibody targeting the allograft inflammatory factor 1 (AIF-1), is a commonly used marker of microglia. All incubation solutions from the blocking serum onward used Tris buffered saline (TBS) with Triton X-100 as the vehicle; all rinses were with TBS. After a hydrogen peroxide treatment and blocking serum, the sections were immunostained overnight at room temperature with the Iba1 primary antibody (ABCam, AB178846) diluted to 1:50,000. We worked with NeuroScience Associates to optimize the protocol for our tissue and antibodies to minimize background noise and settled upon the dilution of 1:50,000 as this provided visualization of intensity differences across regions while minimizing background noise. Vehicle solutions contained Triton X-100 for permeabilization. Following rinses, a biotinylated secondary antibody (Vector, BA-1000) was applied. After further rinses Vector Lab’s ABC solution (avidin–biotin-HRP complex; details in instruction for VECTASTAIN® Elite ABC, Vector, Burlingame, CA) was applied. The sections were again rinsed, then treated with diaminobenzidine tetrahydrochloride and hydrogen peroxide to create a visible reaction product. Following further rinses, the sections were mounted on gelatin coated glass slides and air dried. The slides were dehydrated in alcohols, cleared in xylene and coverslipped.

### Slide identification

Each slide was laser etched with the block number and the stain. Following serial ordering of slides, rostral to caudal for each stain, the slides were numbered by permanent ink in the upper right corner.

### Stereological quantification

**Fig. 2:**
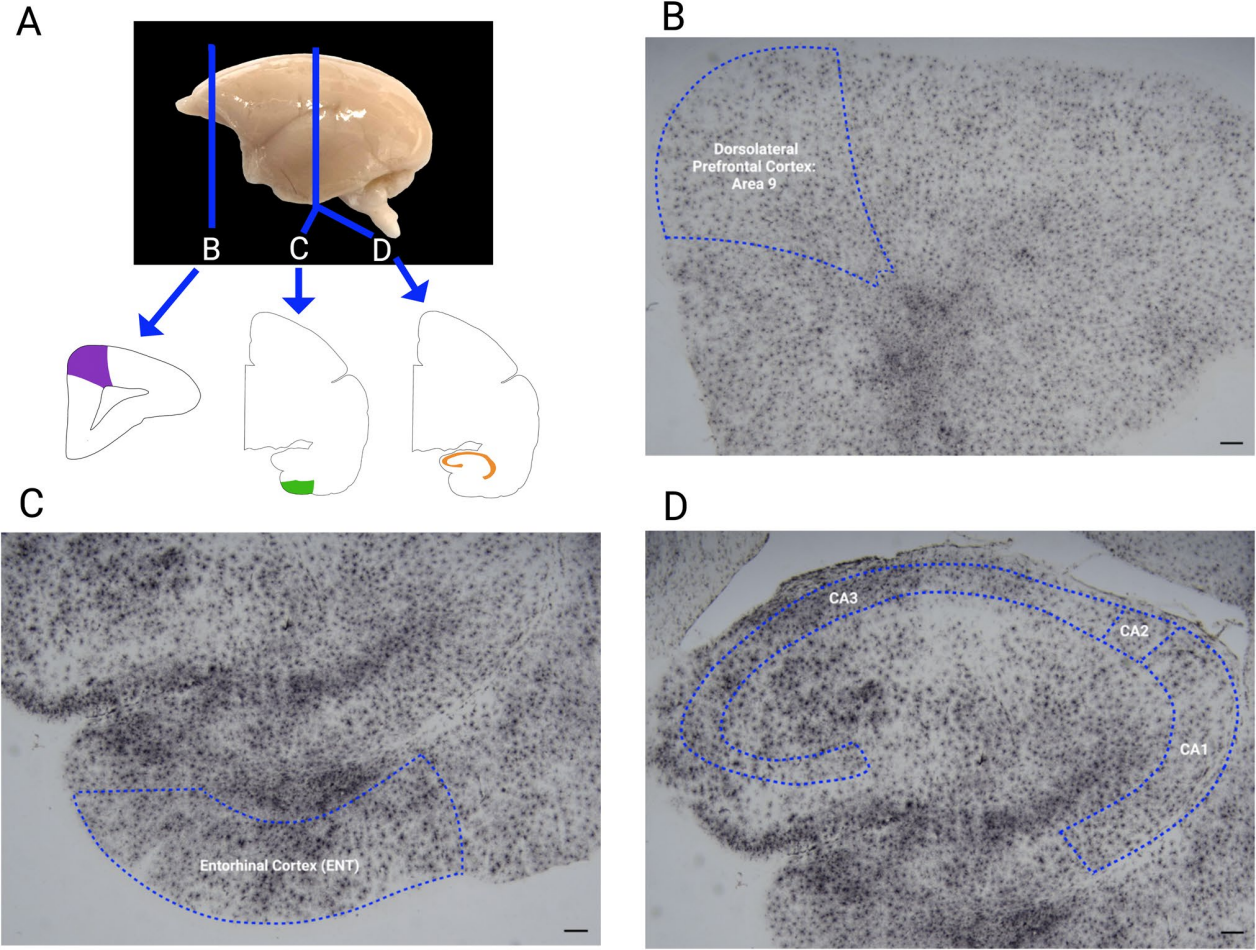
Histological images showing dorsolateral prefrontal cortex (dlPFC), hippocampal regions CA3 and CA1, and entorhinal cortex (ENT) traced from coronal sections in an aged marmoset brain stained for IBa1+ microglia. **A** Lateral view of a marmoset brain illustrating where coronal blocks were obtained. **B** Tracing of dorsolateral prefrontal cortex (A9). **C** ENT located just under the hippocampal formation. **D** Tracing of the CA1 andCA3 fields of the hippocampal formation. Scale bar: 100 μm

Quantitative analyses of Thionin-Nissl and Iba1 were performed using unbiased stereology with a Leica DM6 upright microscope equipped with StereoInvestigator software (Version 2024.2.1, MBF). The Nissl-stained sections were also used to identify regions of interest based on a marmoset brain atlas (Paxinos et al. 2012). Three consecutive slides from the Nissl-stained sections and the Iba1 immunostained sections were chosen from the central portion of area 9 of the dorsolateral prefrontal cortex (dlPFC), CA1 and CA3 hippocampal regions, and entorhinal cortex (Fig. 2). In StereoInvestigator, the optical fractionator workflow for counting numbers was utilized with predetermined sampling parameters collected from previous stereological analysis to quantify neurons and microglia. Stereological parameters, including ROIs, counting frame, grid size, average number of sampling sites, and average CE values are reported in Table 1. Contours were drawn around the regions of interest guided by a marmoset brain atlas for each section under low magnification using a 2.5X objective lens. Once the ROI was traced, a 40× objective lens was used with a 6 µm disector height, a 1 µm guard zone, and section thickness measured at every sampling site. The cortical thickness spanning from layer II to the bottom of layer IV was analyzed for dlPFC and entorhinal cortex. In CA1 and CA3 hippocampal regions, we analyzed the stratum pyramidale. Neurons were identified in Nissl-stained sections based on established morphological criteria, including large soma size, cytoplasmic volume, nuclear morphology, and the presence of a prominent nucleolus. While Nissl staining does not provide molecular specificity, this approach yields neuron-enriched estimates that are widely used in comparative neuroanatomy. The values reported here therefore represent morphologically defined neuronal densities, rather than total cellular densities. Somas of each cell in the counting frame were marked and quantified using specific marker types for each cell type. Analysis of Nissl-stained sections used one marker to label neurons within the counting frame. Analysis of Iba1-stained sections employed four markers to differentially label the somas of the microglia based on their morphological subtype (ramified, intermediate, amoeboid, dystrophic).

**Table 1:** Stereological parameters for the quantification of total neuron density and total microglia counts in Dorsolateral Prefrontal Cortex (dlPFC), Hippocampal Regions CA1 and CA3, and Entorhinal Cortex (ENT)

Marker	Region	Stereology variables	Counting frame (µm)	Disector height (µm)	Avg section thickness (µm)	Average sampling sites	Average density (cells per mm^3^)	Average CE (m = 1)
Nissl	dlPFC	Total Neurons	40 × 40	6	9.55	63 ± 15	215,900 ± 19,356	.05
	CA3				9.50	46 ± 13	163,518 ± 20,812	.07
	CA1				9.74	43 ± 13	127,147 ± 20,173	.08
	ENT				8.56	61 ± 6	140,196 ± 11,942	.06
Iba1	dlPFC	Total Microglia	40 × 40	6	9.44	69 ± 14	15,728 ± 4611	.16
	CA3				9.15	52 ± 13	18,194 ± 4774	.17
	CA1				9.28	42 ± 7	16,084 ± 4806	.20
	ENT				8.69	61 ± 8	16,907 ± 5563	.16

Total microglia counts include four subtypes: ramified, intermediate, amoeboid, and dystrophic. The coefficient of error (CE) is estimated based on Gunderson m = 1 formula (Gundersen et al. 1999)

Once the optical fractionator workflow was completed for all three sections of the ROI, cell densities (cells per mm^3^) were calculated as the cell types estimated population divided by the volume of the region of interest. The coefficient of error (CE) of cell counts was calculated through the Gunderson CE m = 1 curve (Gundersen et al. 1999), which describes the variance within the systematic random sampling across sections (CE < 0.1 is typically considered acceptable).

It should be noted that differences in neuronal density values between the present study and some prior reports (i.e., Atapour et al. 2019) likely reflect methodological differences rather than biological discrepancies. NeuN-based estimates derived from 2D image profile counts are known to yield lower absolute densities than Nissl-based optical stereological approaches, due both to incomplete NeuN labeling of neuronal populations and to assumptions regarding effective section thickness. Consistent with this interpretation, prior work comparing NeuN and Nissl counts within the same tissue has shown higher absolute values with Nissl staining but strong correlations across regions (Atapour et al. 2019), indicating that relative patterns of variation are preserved despite differences in absolute magnitude.

### Classifying microglia morphology

**Fig. 3:**
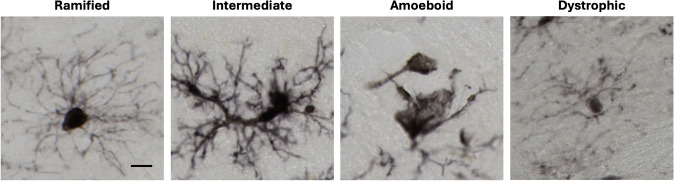
Histological images of the four microglia morphologies quantified in this study. **A** Ramified microglia exhibit small, rounded cell somas with long, highly ramified processes that survey the surrounding environment in a resting state. **B** Microglia activate into an anti-inflammatory, intermediate state with a hypertrophic soma, and thickening of the ramified processes. **C** Upon chronic activation, microglia take on an amoeboid shape with an enlarged, irregularly shaped somas and a loss of processes. **D** Dystrophic microglia display spheroid swellings, and/or cytoplasmic fragmentation with short, beaded, torturous processes that become dysfunctional in a senescent state. Scale bar: 10 μm

To classify the reactive state of microglia and potential phagocytic activity, microglia densities for four morphological subtypes were quantified—ramified, intermediate, amoeboid, and dystrophic. Ramified microglia morphology includes a small, rounded cell soma with long, highly branched processes. Intermediate microglia morphology includes an enlarged, irregularly shaped cell soma with shorter, thicker, and less branched processes. Amoeboid microglia have a rounded, highly enlarged cell soma with little to no processes left. Dystrophic microglia morphology includes spheroid swellings and/or cytoplasmic fragmentation with beaded, shortened, tortuous processes (Kettenmann et al. 2011; Rodriguez-Callejas et al. 2019). Figure 3 illustrates these microglial morphologies.

### Data analysis

We were primarily interested in whether microglial morphologies were associated with age and/or sex in dlPFC, CA1, CA3, and ENT. Statistical analysis was conducted in R using the packages *compositions* v2.0–8 (Boodaart et al. 2024) and *stats* v4.4.1. One-way ANOVA was performed to examine whether neuron or activated microglial density varied across dlPFC, CA1, CA3, and ENT. Pearson correlation analysis was used to quantify the relationship between age and microglial proportions. Centered log ratio (CLR) transformations were performed on the four microglia morphology proportions prior to the analysis. The variable age was centered to avoid multicollinearity in the models. MANOVA was used to assess the effects of age and sex and the interaction between age and sex on the proportions within each region. Alpha was set at 0.05.

## Results

The data for neuron density and proportions of microglial phenotypes can be found in Supplemental Materials.

**Fig. 4:**
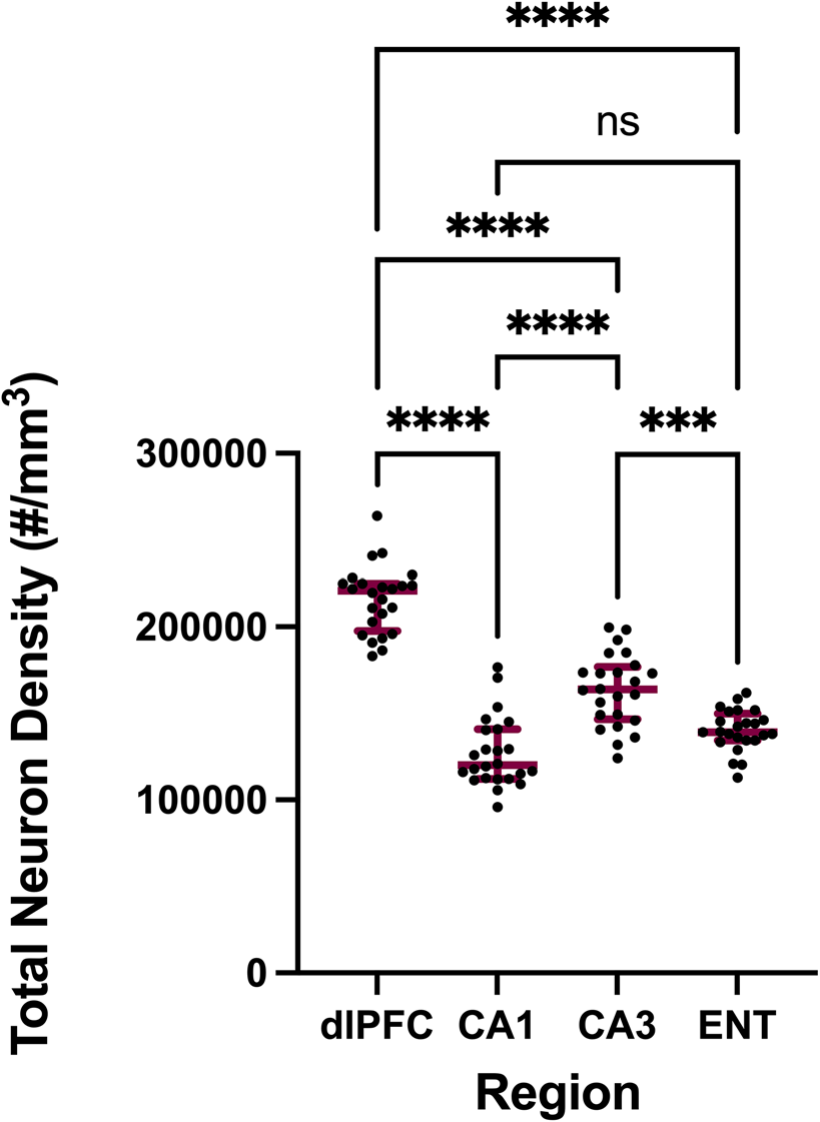
Neuron density (cells per mm^3^) in dorsolateral prefrontal cortex (dlPFC), hippocampal regions CA1 and CA3, and entorhinal cortex (ENT). A significantly greater density of neurons was found in dlPFC compared to all other regions. CA3 had significantly higher neuron density compared to CA1 and ENT. Bars represent median and interquartile range. ****p < 0.0001; ***p < 0.001

The average neuron density for each region is provided in Table 1. Neuron density varied significantly across the regions of interest examined in this study (*F*(3, 92) = 108.4, *p* < 0.0001), with dlPFC exhibiting significantly higher neuron density compared to all other regions (*p* < 0.0001). CA3 had significantly higher neuron density compared to CA1 (*p* < 0.0001) and ENT (*p* = 0.0002) (Fig. 4). Neuron density showed a weak, positive correlation with age in CA1 (*r*(22) = 0.51, *p* = 0.01). There were no other associations between age and neuron density.

**Fig. 5:**
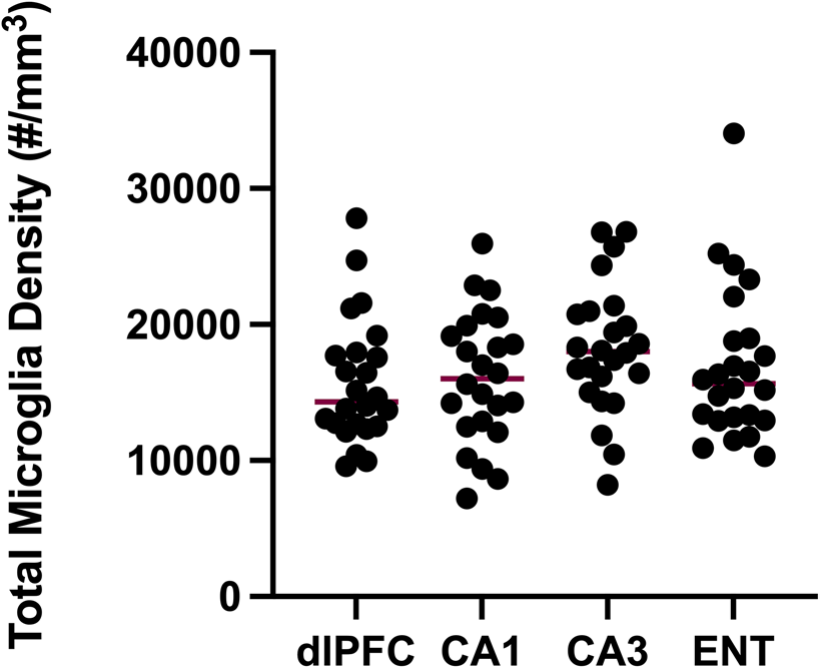
Total microglia density (cells per mm^3^) in dorsolateral prefrontal cortex (dlPFC), hippocampal regions CA1 and CA3, and entorhinal cortex (ENT)

The average total microglial density for each region of interest is provided in Table 1**;** the density of total microglia per region is presented in Fig. 5. Across this cohort of aged common marmosets, all four microglial phenotypes were observed in all four regions of interest, in all individuals. The density of activated microglia (combining ramified, intermediate, and amoeboid) did not differ across the dlPFC, CA1, CA3, and ENT in our sample of aged marmosets (*F*(3, 92) = 0.675, *p* = 0.57).

Age had a significant effect on the proportion of ramified microglia in dlPFC (*F*(1,20) = 7.64, *p* = 0.012), CA1 (*F*(1,20) = 14.52, *p* = 0.001), and CA3 (*F*(1,20) = 10.27, *p* = 0.004). Age also had a significant effect on the proportion of dystrophic microglia in all regions (dlPFC: *F*(1,20) = 7.65, *p* = 0.012; CA1: *F*(1,20) = 4.43, *p* = 0.048; CA3: *F*(1,20) = 20.53, *p* < 0.001; and ENT: *F*(1,20) = 7.85, *p* = 0.011).

The proportion of dystrophic microglia in CA1 was significantly affected by sex (*F*(1,20) = 5.20, *p* = 0.03); males had a significantly higher proportion of dystrophic microglia than females. Sex did not significantly affect the proportion of microglia morphologies in any other brain region.

The interaction between age and sex significantly affected the proportion of intermediate microglia in ENT (*F*(1,20) = 21.83, *p* < 0.001). The proportion of intermediate microglia in females decreased with age, whereas the proportion of intermediate microglia increased with age for males.

**Fig. 6:**
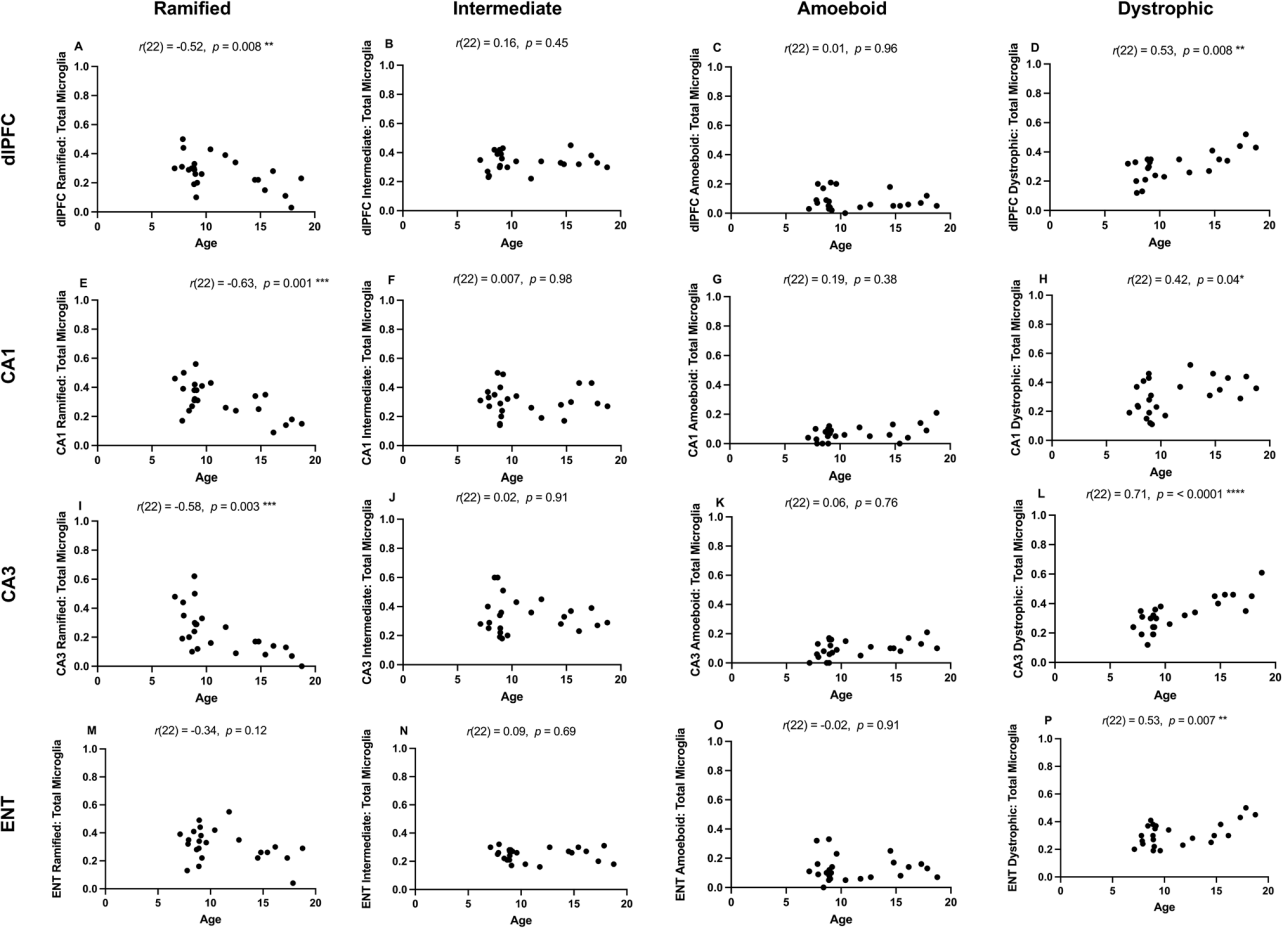
Associations between age and microglia morphology in dorsolateral prefrontal cortex (dlPFC), hippocampal regions CA1 and CA3, and entorhinal cortex (ENT). The proportion of ramified microglia showed a significant negative correlation with age in dlPFC, CA1 and CA3. The proportion of dystrophic microglia showed a significant positive correlation with age in all four regions examined. *p < 0.05; **p < 0.01; ***p < 0.001

Results from the Pearson’s correlational analyses are illustrated in Fig. 6. The proportion of ramified microglia showed a significant negative correlation with age in the dlPFC, CA1, and CA3, indicating a decline in surveillant microglial states with advancing age. In contrast, the proportion of dystrophic microglia increased significantly with age in all four regions examined (dlPFC, CA1, CA3, and ENT), suggesting an age-related shift toward degenerative microglial phenotypes.

## Discussion

We report on changes in the proportion of microglial morphologies from middle-aged to older adults in a relatively large sample of common marmosets. These data indicate a distinct pattern of microglial morphological changes with age in dlPFC, CA1, CA3, and ENT: the proportion of ramified microglia decreased, which was accompanied by an increase in the proportion of dystrophic microglia. This indicates an age-related decline in the microglia’s ability to perform their essential surveillance and immune functions in the central nervous system. The increase in proportion of dystrophic microglia in brain regions associated with cognitive performance underscores their role as a potential mechanism underlying neurodegenerative diseases associated with brain aging. Sex differences were found in only one brain region, CA1, with males having a significantly higher proportion of dystrophic microglia than females. A significant interaction between age and sex was found for the proportion of intermediate microglia in ENT. Females had a decrease in the proportion of intermediate microglia with age, whereas the proportion of intermediate microglia increased with age for males.

Neuronal density is highly variable across cortical areas and layers among primates. The range in neuronal density reflects important functional and structural specialization of different cortical areas. The primary visual cortex (V1) consistently exhibits the highest neuronal density. Areas with lower neuronal density, such as allocortical regions, tend to have larger neurons with more expansive dendritic trees that take up space between somas, reflecting specializations for integrative, higher-order cognitive functions. In marmoset cortex, the neuronal density varies widely across different areas, with the highest density in the primary visual cortex (V1) and allocortical areas having lower densities (Atapour et al. 2019). Neuronal density also varies systematically across layers of the superior colliculus in common marmosets, decreasing from superficial to deeper strata (Chong et al. 2022). Our results, finding cortical region dlPFC had significantly higher neuron density than areas of the hippocampal formation (CA1, CA3, and ENT), suggests more computational capacity in this key brain structure for higher-order cognitive processing. A surprising finding concerned the positive association between neuron density and age in CA1, as studies have indicated neuron density in CA1 remains relatively stable with normal aging (Lister and Barnes 2009). The increase in CA1 neuron density with age observed in this cohort could be due to a corresponding increase in glial cells and/or selective hippocampal atrophy, rather than a true increase in total neuron number.

Prior studies examining changes in microglial morphologies with age in nonhuman primates have reported increased microglial activation and densities. Edler et al. (2018) examined changes in microglial morphology in aged chimpanzees, in several brain regions associated with cognition and Alzheimer’s disease, including the prefrontal cortex and hippocampal regions CA1 and CA3. In their analyses, they considered ramified, intermediate, and amoeboid as “activated microglia” and found CA3 had a significantly higher density of activated microglia than CA1. Rodriguez-Callejas et al. (2016) reported an increase in intermediate microglia in the hippocampus and cortex of old male marmosets. We did not find significant differences in the density of activated microglia across the dlPFC, CA1, CA3, and ENT in our sample of aged marmosets. However, we did detect a significant interaction between age and sex for the proportion of intermediate microglia in the ENT. Females displayed a decrease with age, whereas the proportion of intermediate microglia increased with age for males. One important consideration is that there is no consistent classification of morphologies that are being quantified across studies, particularly with regards to whether dystrophic microglia are considered their own phenotype. For example, Edler et al. (2018, 2021) only quantified ramified, intermediate, and amoeboid microglia in chimpanzees. Freire-Cobo et al. (2023) quantified only two morphologies in their sample of marmosets: resting and intermediate. However, Rodriquez-Callejas et al. (2016, 2019) included dystrophic microglia in their studies of marmosets and tree shrews (*Tupaia belangeri*), reporting an increase in dystrophic microglia in the brains of old individuals. We classified and quantified four morphological subtypes—ramified, intermediate, amoeboid, and dystrophic—to provide a more comprehensive picture of how microglial morphologies change with age.

How these changes in microglial morphologies are associated with age-related cognitive decline in marmosets is unclear at present. A recent study suggested cognitively impaired aged marmosets showed signs of neurodegeneration and accelerated brain aging, including an increase in intermediate microglia compared to ramified microglia in dlPFC (Freire-Cobo et al. 2023). Rothwell et al. 2022 reported that while male and female marmosets displayed declines in performance on discrimination and reversal learning tasks, females showed an earlier and greater decline than males. While corresponding data on neurodegeneration were not provided by Rothwell et al. (2022), the observed sex difference in cognitive function would suggest females would have a greater amount of activated and dystrophic microglia in hippocampal regions compared to males. While we did not obtain corresponding cognitive data from the sample of marmosets in the current study, our results, of males having a significantly higher proportion of dystrophic microglia than females in CA1, contrast with what would be predicted by Rothwell et al.’s results. Additional work is needed to explore sex differences in cognitive aging and associated neurodegenerative change.

Taken together, our results indicate brain aging in marmosets from middle-age to older adults is characterized by an increase in dystrophic microglia, and age-related changes in microglial morphology manifest differently in male and female marmosets in hippocampal regions. These data add to the growing body of evidence indicating that older marmosets develop morphological changes reflecting neurodegeneration and functional impairment. However, one important limitation of the current study is that we did not include young adult marmoset samples in our data set as a comparison. Therefore, we cannot unequivocally state that the observed changes in microglia morphologies were due to age. The inclusion of young adult marmosets will thus be an important area for future investigations. Future studies investigating functional changes in microglia associated with aging and linking to cognition will provide critical insight into the mechanisms by which microglial senescence contributes to cognitive impairment. Additionally, the inclusion of inflammatory biomarker data (via plasma or CSF) will provide critical insight into whether the observed morphological changes are a result of microglia dysfunction or overall elevated inflammation.

## Supplementary Information

Below is the link to the electronic supplementary material.

Supplementary file1 (DOCX 16 kb)

Supplementary file2 (DOCX 20 kb)

## Data Availability

The datasets generated during and/or analyzed during the current study are available from the corresponding author upon request.
