# Supplementary material for: Changes in microglial morphologies during brain aging in common marmosets
Source: Brain Struct Funct. 2026 Feb 17;231(2):27. doi: 10.1007/s00429-026-03082-z (PMC12913261; doi:10.1007/s00429-026-03082-z)
Supplement: Supplementary file 1 — Supplementary file1 (DOCX 16 kb) [file 429_2026_3082_MOESM1_ESM.docx]

**Table 3: Neuron Density (cells per mm^3^) in Dorsolateral Prefrontal Cortex (dlPFC), Hippocampal Regions CA1 and CA3, and Entorhinal Cortex (ENT).**

____________________________________________________________________________________________________________

**Sex Age dlPFC CA1 CA3 ENT**

F 7.1 219,603 111,444 173,131 138,086

F 7.9 223,846 129,045 164,237 144,221

F 7.9 183,073 111,949 149,158 134,411

F 8.9 210,915 119,390 140,667 151,160

F 8.9 202,965 128,364 149,416 139,197

F 9.0 222,848 116,512 199,590 139,269

F 9.2 193,251 146,670 163,439 146,074

F 10.4 240,818 112,050 198,428 136,062

F 14.5 224,934 116,033 173,605 137,484

F 17.3 229,938 145,060 159,844 145,487

F 18.8 207,609 117,908 160,807 144,367

M 7.8 190,757 129,431 131,905 153,953

M 8.4 228,002 105,649 145,783 151,829

M 8.7 186,314 109,114 192,433 128,815

M 8.9 263,965 115,220 142,393 134,476

M 8.9 195,810 120,992 184,811 133,390

M 9.1 211,161 125,938 136,119 151,857

M 9.6 221,993 112,613 168,332 120,865

M 11.8 221,927 95,969 124,123 161,830

M 12.7 195,042 153,672 185,196 120,286

M 14.8 223,669 140,935 177,795 138,108

M 15.4 224,931 176,743 173,729 142,360

M 16.2 215,850 170,511 173,216 158,377

M 17.9 242,347 140,311 156,254 112,763
