# Supplementary material for: Changes in microglial morphologies during brain aging in common marmosets
Source: Brain Struct Funct. 2026 Feb 17;231(2):27. doi: 10.1007/s00429-026-03082-z (PMC12913261; doi:10.1007/s00429-026-03082-z)
Supplement: Supplementary file 2 — Supplementary file2 (DOCX 20 kb) [file 429_2026_3082_MOESM2_ESM.docx]

**Table 4. Ramified, Intermediate, Amoeboid, Dystrophic, and Total Microglia Densities (cells per mm3) in regions dlPFC, CA1, CA3, and ENT in 24 aged marmosets.**

dlPFC CA1

Sex Age Total Ramified Intermediate Amoeboid Dystrophic Total Ramified Intermediate Amoeboid Dystrophic

F 7.1 13,707 4,075 4,816 370 4,446 12,089 5,579 3,720 465 2,325

F 7.9 10,400 5,200 2,427 693 2,080 22,877 9,016 7,629 694 5,548

F 7.9 12,678 5,578 3,043 2,536 1,521 14,063 7,031 3,750 0 3,281

F 8.9 12,500 3,750 3,750 625 4,375 12,480 4,754 1,783 594 5,349

F 8.9 16,558 5,407 5,069 1,352 4,731 14,220 4,550 5,688 0 3,982

F 9.0 12,301 3,209 4,813 535 3,744 14,291 8,003 2,858 1,715 1,715

F 9.18 17,583 3,440 7,645 382 6,116 19,900 6,254 9,666 1,706 2,274

F 10.4 16,454 7,002 5,601 0 3,851 22,511 9,648 7,718 1,286 3,859

F 14.5 21,570 4,728 7,091 3,841 5,909 20,524 7,055 5,772 1,283 6,414

F 17.3 17,944 1,994 6,779 1,196 7,975 16,433 2,348 7,043 2,348 4,695

F 18.8 14,010 3,184 4,139 637 6,050 18,322 2,776 4,997 3,886 6,663

M 7.8 15,153 4,735 4,025 1,421 4,972 19,158 3,271 7,009 1,869 7,009

M 8.4 9,940 2,899 4,142 1,657 1,243 7,216 1,698 2,547 0 2,971

M 8.7 9,583 2,904 3,775 871 2,033 17,002 4,578 8,501 1,308 2,616

M 8.9 21,197 3,944 8,873 986 7,394 9,399 2,892 1,446 723 4,338

M 8.9 12,109 3,460 4,844 346 3,460 14,868 6,235 4,316 1,439 2,878

M 9.1 27,822 2,650 9,937 5,962 9,274 15,620 5,925 3,770 1,077 4,848

M 9.6 17,725 4,624 5,395 3,468 4,239 10,198 4,172 3,245 464 2,318

M 11.8 13,829 5,411 3,006 601 4,810 8,659 2,279 2,279 911 3,190

M 12.7 12,753 4,331 4,331 722 3,369 18,030 4,293 3,434 859 9,444

M 14.8 14,634 3,212 4,640 714 6,068 18,542 4,635 3,090 2,318 8,498

M 15.4 19,196 2,879 8,638 960 6,719 12,870 4,476 3,917 0 4,476

M 16.2 13,095 3,667 4,190 786 4,452 20,790 1,808 9,039 904 9,039

M 17.9 24,730 773 8,115 3,091 12,752 25,938 4,577 7,629 2,289 11,443

CA3 ENT

Sex Age Total Ramified Intermediate Amoeboid Dystrophic Total Ramified Intermediate Amoeboid Dystrophic

F 7.1 16,429 7,886 4,600 0 3,943 15,309 5,915 4,523 1,740 3,131

F 7.9 10,437 4,566 2,609 1,305 1,957 16,543 5,224 4,353 2,612 4,353

F 7.9 17,930 6,328 5,273 703 5,625 11,483 4,053 3,715 1,013 2,702

F 8.9 16,180 3,906 5,579 2,790 3,906 25,208 7,389 6,954 1,304 9,562

F 8.9 19,873 5,908 4,297 3,223 6,445 12,914 6,283 3,141 1,047 2,443

F 9.0 18,594 5,313 6,641 2,214 4,427 10,303 4,508 2,898 644 2,254

F 9.2 26,831 3,111 13,610 1,944 8,166 23,318 5,182 6,292 3,331 8,513

F 10.4 25,713 4,159 10,966 3,781 6,806 13,419 5,650 2,472 706 4,591

F 14.5 16,847 2,905 4,647 1,743 7,552 18,967 4,091 5,207 4,835 4,835

F 17.3 20,765 2,679 8,038 2,679 7,368 22,066 4,797 4,317 3,538 9,594

F 18.8 17,392 0 5,049 1,683 10,660 18,769 5,460 3,413 1,365 8,532

M 7.8 18,328 3,436 7,255 1,145 6,491 34,036 4,314 8,629 11,026 10,067

M 8.4 11,834 2,367 7,101 947 1,420 13,188 5,373 2,931 0 4,884

M 8.7 14,209 1,421 8,525 0 4,263 10,904 3,008 2,256 1,128 4,512

M 8.9 8,229 5,143 1,543 0 1,543 24,398 4,006 5,098 8,011 7,283

M 8.9 14,346 7,173 3,587 897 2,690 14,734 5,031 3,953 1,797 3,953

M 9.1 20,793 6,101 3,813 3,432 7,626 16,956 6,359 2,967 1,696 5,935

M 9.6 16,733 5,578 3,347 1,487 6,321 11,751 3,826 3,006 2,733 2,186

M 11.8 15,007 4,093 5,457 682 4,775 12,962 7,108 2,091 836 2,927

M 12.7 19,423 1,766 8,829 2,207 6,621 15,966 5,588 4,790 1,197 4,391

M 14.8 18,092 3,015 6,031 1,723 7,323 17,683 4,671 4,671 3,003 5,338

M 15.4 24,341 2,057 8,914 2,057 11,313 13,354 3,428 3,970 1,083 5,053

M 16.2 21,374 3,053 4,885 3,664 9,771 15,190 4,488 4,143 2,071 4,488

M 17.8 26,778 1,785 7,141 5,713 12,139 16,347 711 5,131 2,132 8,173
